# What Can State Medical Boards Do to Effectively Address Serious Ethical Violations?

**DOI:** 10.1017/jme.2024.6

**Published:** 2023

**Authors:** Tristan McIntosh, Elizabeth Pendo, Heidi A. Walsh, Kari A. Baldwin, Patricia King, Emily E. Anderson, Catherine V. Caldicott, Jeffrey D. Carter, Sandra H. Johnson, Katherine Mathews, William A. Norcross, Dana C. Shaffer, James M. DuBois

**Affiliations:** 1: BIOETHICS RESEARCH CENTER, WASHINGTON UNIVERSITY SCHOOL OF MEDICINE, ST. LOUIS, MO, USA; 2: CENTER FOR HEALTH LAW STUDIES, SAINT LOUIS UNIVERSITY SCHOOL OF LAW, SAINT LOUIS, MO, USA; 3: LARNER COLLEGE OF MEDICINE, UNIVERSITY OF VERMONT, BURLINGTON, VT, USA; 4: LOYOLA UNIVERSITY CHICAGO STRITCH SCHOOL OF MEDICINE, MAYWOOD, IL, USA; 5: PBI EDUCATION, JACKSONVILLE, FL, USA; 6: MISSOURI BOARD OF REGISTRATION FOR THE HEALING ARTS, JEFFERSON CITY, MO, USA; 7: DEPARTMENT OF OBSTETRICS, GYNECOLOGY, AND WOMEN’S HEALTH, SAINT LOUIS UNIVERSITY SCHOOL OF MEDICINE, ST. LOUIS, MO, USA; 8: DIVISION OF FAMILY MEDICINE, UC SAN DIEGO SCHOOL OF MEDICINE, LA JOLLA, CA; 9: UNIVERSITY OF PIKEVILLE KENTUCKY COLLEGE OF OSTEOPATHIC MEDICINE; 10: KENTUCKY BOARD OF MEDICAL LICENSURE, LOUISVILLE, KY, USA

**Keywords:** State Medical Boards, Egregious Wrongdoing, Physician Misconduct, Policy Change, Protecting Patients

## Abstract

State Medical Boards (SMBs) can take severe disciplinary actions (e.g., license revocation or suspension) against physicians who commit egregious wrongdoing in order to protect the public. However, there is noteworthy variability in the extent to which SMBs impose severe disciplinary action. In this manuscript, we present and synthesize a subset of 11 recommendations based on findings from our team’s larger consensus-building project that identified a list of 56 policies and legal provisions SMBs can use to better protect patients from egregious wrongdoing by physicians.

State Medical Boards (SMBs) have the authority to take severe disciplinary measures against physicians when necessary to protect the public. Such measures include, but are not limited to, revoking or suspending a physician’s license to practice medicine. About 0.5% of physicians are subject to board disciplinary actions annually, and 0.1% of these involve severe disciplinary actions — a rate similar to annual breast cancer diagnoses and much greater than annual HIV diagnoses.^1^ Severe disciplinary actions are appropriate when physicians have engaged in egregious wrongdoing that directly harms patients, which includes sexually abusing patients, performing unnecessary surgeries for financial gain, or unlawfully prescribing controlled substances.^2^ SMBs vary widely in their use of severe disciplinary action.^3^ Some board characteristics are associated with higher rates of severe disciplinary action, including adequate SMB budget and staffing, independence from regional government, and presence of board members who are not physicians.^4^

Failures to use severe disciplinary actions to protect the public have attracted significant attention. For example, Dr. Farid Fata, an oncologist in Michigan, purposely misdiagnosed patients with cancer and prescribed them unnecessary chemotherapy and radiation therapy, causing undue pain and suffering.^5^ In 2010, an oncology nurse suspected Fata was giving patients drugs needlessly so he could bill their insurance. She informed the Michigan board that patients were being harmed. She did not hear back until a year later when she received a formal letter relaying that the state found no wrongdoing. The investigation was closed.^6^ Fata continued to harm his patients for two more years when his office manager filed a whistleblower lawsuit in 2013. Federal authorities investigated and shut down his practice. His license was suspended, and he was sentenced to 45 years in prison.^7^

In another case in 2001, a patient contacted the police alleging that Philip Leonard, a board certified neurologist practicing in Texas, had rubbed his genitals against her and touched her inappropriately.^8^ Other women started coming forward with similar allegations. Leonard was reported to the Texas Medical Board in May 2002.^9^ In total, Leonard was accused of engaging in sexual misconduct with at least 17 patients and arrested for public lewdness involving two patients.^10^ Leonard’s license was temporarily suspended on March 8th, 2003.^11^ One patient declined requests from the county attorney’s office to testify in a criminal case because of how she was treated by Leonard’s attorney at the board’s public hearing.^12^ Though several women reported the misconduct, only one case was ever prosecuted. There was a lack of forensic evidence and Leonard was acquitted by a jury.^13^ Another criminal case against him was resolved through a plea agreement.^14^ In December 2004, the board restricted Leonard’s license for 10 years.^15^ The restrictions included that he could not have direct or indirect contact with female patients.^16^ The former board president reported that the Board’s limited budget prevented them from hiring expert witnesses and motivated the board to avoid an appeal, which would be costly. Despite his past history of serious sexual misconduct complaints Leonard was allowed by the Texas State Medical Board to resume treating female patients in 2014.^17^ Leonard was accused of similar sexual misconduct by a male patient who, after leaving Leonard’s office, called the police to report that the doctor touched his genitals and made inappropriate comments. Despite the past history of complaints, the police did not investigate. The patient then made two complaints to the board in 2015, and a formal complaint against the doctor was filed in August of 2016.^18^ In 2018, Leonard finally lost his medical license.^19^

Beyond these individual instances, a study of 280 cases of egregious wrongdoing in medicine found that nearly all cases involved repeated instances of intentional wrongdoing (97%).^20^ Another study found that about 20% of physicians receiving severe sanctions by boards are repeat offenders.^21^ Perhaps more surprisingly, in cases of severe wrongdoing, depending on the specific nature of the violation, anywhere from 20% - 39% of physicians resumed medical practice at some point after being investigated, sometimes after relocating to a new state.^22^

A recent Delphi panel consensus-building project aimed to identify policies, practices, and legal provisions needed for SMBs to respond to egregious wrongdoing by physicians in a timely and effective manner.^23^ Panelists included 40 individuals (e.g., physician members, legal counsel, public members, executive staff) from about half of the 71 SMBs in the United States and its territories. Panelists were recruited nationally and represented boards of varying sizes and with different amounts of resources. Panelists were asked to describe any particularly effective or innovative practices, policies, and legal provisions that their board currently has to protect the public from egregious wrongdoing; they were also asked to describe practices, policies, and legal provisions not currently in place but that they believed would better protect the public if implemented. Through a series of surveys, panelists rated the importance of each recommendation. The panel produced a list of 56 high-consensus resources, policies, and practices, which are described in a separate paper.^24^

In this paper, we explore a particular subset of these recommendations within each of these clusters: those that are within the power of most boards to enact without the need for changes to the state legislative framework, which can be slow and difficult to change. To illustrate, the recommendation to reclassify all sexual offenses by physicians as felony offenses, subjecting them to mandatory reporting would significantly disrupt state systems of criminal and civil laws addressing sexual offenses and misconduct, making this type of strategy more challenging to implement. We also focus on recommendations that are likely to have the greatest impact in protecting patients from egregious wrongdoing by physicians. We suggest model state statutory provisions elsewhere.

Based on input from members of the project’s advisory board — comprised of past Federation of State Medical Boards (FSMB) leadership, leading health lawyers, leaders of physician remediation training programs, leaders in healthcare ethics, patient advocates, and members of SMBs — the recommendations were clustered into five topics by the research team. These topics include: 1) board composition, characteristics, and training, 2) board website, outreach, and education, 3) internal board operations and investigations, 4) improved coordination and information sharing among stakeholders, and 5) licensing and disciplinary considerations.

In this paper, we explore a particular subset of these recommendations within each of these clusters: those that are within the power of most boards to enact without the need for changes to the state legislative framework, which can be slow and difficult to change. To illustrate, the recommendation to reclassify all sexual offenses by physicians as felony offenses, subjecting them to mandatory reporting would significantly disrupt state systems of criminal and civil laws addressing sexual offenses and misconduct, making this type of strategy more challenging to implement. We also focus on recommendations that are likely to have the greatest impact in protecting patients from egregious wrongdoing by physicians. We suggest model state statutory provisions elsewhere.^25^ Some SMBs regulate physician and non-physician licensees, but we focus on policies that are likely to curb egregious wrongdoing by physicians specifically. We did not include recommendations from the consensus panel that we deemed impractical or infeasible (e.g., recommendations that conflict with certain legal principles), or that focus on less substantial matters unlikely to protect patients from harm (e.g., minor professionalism violations).

While SMBs are governed by medical practice acts and other state laws, they can autonomously adopt certain board policies and practices, especially as these policies and practices relate to medical practice (i.e., physician licensing and discipline).^26^ In what follows, we present the five clusters of select stakeholder-informed recommended policies that SMBs may be able to adopt without the need for legislation or external government action. For each of the 11 recommendations, we explain how the policy benefits SMBs in their efforts to protect patients and identify factors for boards to consider as they implement each policy. Synthesis of these 11 recommendations is informed by past research and first-hand experiences of advisory board members (all co-authors of this manuscript) who have an intimate understanding of these issues. The wording of recommendations presented in this manuscript differs slightly compared to wording of recommendations in the Delphi panel manuscript in light of reflection and refinement based on feedback from expert stakeholders about putting these recommendations into action.

## Cluster 1: Board composition, characteristics, and training

*Recommendation 1: Diversify boards to include effective gender, racial, and community (e.g., non-physician public members) representation.*

The FSMB has called for diversity in SMB membership in terms of gender, race, ethnicity, and other characteristics to reduce the impact of implicit bias and cultivate balanced discussion and decisions.^27^ There are several reasons to support increased gender, racial and ethnic, and other types of diversity in SMB membership, including improvement of board function and decision-making.^28^ Research from other sectors has demonstrated that diverse teams perform better and achieve better outcomes than homogenous teams.^29^ Studies indicate that diverse teams can contribute to improved and more accurate group thinking, including a more careful and deliberative focus on available facts and consideration of alternate viewpoints.^30^ Diversity also presents advantages to group decision-making through improved information exchange.^31^ We recommend model language for state statutes that mandate gender, racial, and ethnic diversity in a separate paper.^32^ Here, we focus on strategies to increase diversity that boards can adopt and implement without changes to state statutes.

In many states, board members are appointed by the governor or a nominating committee, a process that relies on nominations from state medical organizations, boards, and other sources.^33^ Boards should prioritize diversity when recommending or nominating candidates, as well as in whatever advisory capacity they may have regarding member selection. For example, boards could liaise with and communicate diversity-related goals to staff in governors’ offices and members of the National Governors Association. Boards should also prioritize diversity in hiring board staff who perform important roles in screening and investigating complaints made to the board about physicians.^34^ In many instances, diversifying staff will be the only option directly available to boards for increasing diversity. Focusing on diversifying staff who support the board or investigate cases may be a good first step while pursuing changes to state statutes to increase diversity of appointed board members. In some states, state medical societies have a greater say in the nomination process of board members. In these states, partnerships between SMBs and state medical societies may be a promising avenue for cultivating change.

The FSMB recommends that 25% of a board’s members should be public members.^35^ As an element of promoting diversity and inclusion, including a critical mass of public members on SMBs gives the public an effective voice. Public members provide a perspective unique from those of physician members; having an appropriate balance of perspectives from different stakeholder groups can surface and address unconscious biases that may emerge in a self-regulatory environment.^36^

Public members on boards should represent other forms of diversity, but other forms of diversity should not be limited to public members. Having diverse board members and staff has the potential to reduce implicit bias throughout the investigation and disciplinary processes. A diverse board may also be more likely to advocate for impacting broader health equity issues. Beyond board makeup, boards should also strongly consider committing themselves to a professional development process that includes exposure to racial and gender issues in medical care (e.g., disparities in access, treatment, and health outcomes; employment, education, and training of underrepresented providers) and in their own decision-making. Gender diversity and implicit bias training may be of particular importance for investigations of sexual misconduct, which boards commonly investigate.^37^

Implicit bias training can also be provided to board members to help minimize implicit bias and promote responsibility for practices that support diversity and inclusion within and between boards.^38^ A universal training program for new board members could help members work more effectively together and eliminate inter-state differences in duties, responsibilities, and processing. The FSMB may wish to take a leadership role in such standardization, just as it has made recommendations regarding standardizing other board activities (e.g., educational resources that help address implicit bias in medical regulation).^39^ While training programs for board members are likely to be an important part of the solution, structural and policy changes that support diversity and inclusion on boards are also needed.

## Cluster 2: Board Website, Outreach, and Education

*Recommendation 2a: Update SMB websites to include the following educational elements for patients and the public at large: 1) investigation processes, 2) state laws and policies on sexual misconduct, 3) findings from disciplinary hearings, and 4) reporting mechanisms.*

*Recommendation 2b: Market the SMB’s purpose via social media, professional organizations, and liaising with hospitals and other relevant groups.*

SMBs play a critical role in protecting patients from harm, protecting public interest, and regulating the medical profession. But boards can only act on those complaints they receive. Most of the public is unaware that SMBs exist and that boards are one of the best resources for filing a complaint about a physician’s conduct.^40^ This lack of awareness makes it less likely that harmful physicians will be reported to boards. SMBs can leverage online platforms (e.g., board websites or social media) and relationships with other professional organizations and healthcare institutions to publicize and raise awareness about the purpose and function of SMBs.

Recently, the FSMB championed an initiative to develop the website docinfo.org, which provides publicly available information about U.S. physicians’ medical license history and disciplinary actions taken against physicians by SMBs. Boards can work with healthcare institutions to develop marketing materials about docinfo.org and the purpose of SMBs and distribute these materials to patients. Other relevant stakeholder- and evidence-informed resources for patients are also available (e.g., www.preventingsexabuse.org and www.beforeyourvisit.org) and can be distributed by boards and healthcare institutions to educate patients about receiving appropriate care and responding to inappropriate care.^41^ Patients who do not have internet connectivity will need other ways to receive these resources, such as through materials distributed by community centers or public assistance programs.

Being explicit and intentional in communicating this information to patients can cultivate greater public awareness. Ensuring robust and consistent complaint categories can further support patient education and allow for better data collection. Having a thorough list of reportable behaviors (e.g., including complaint categories for various forms of egregious wrongdoing, racism, and bias) sends a message to the public about what issues the board thinks are important to address. When patients understand what behaviors warrant reporting and the role that boards play in investigating and disciplining cases of physician wrongdoing, they may feel more confident about reporting and how to report; they may also report more consistently and appropriately. Through the board website, social media, and other online platforms, a board can empower patients with easy-to-find information about its function, provide details about how to report suspected wrongdoing, and direct patients to other pertinent information.^42^ For example, the public should be educated about the disturbing prevalence of physician sexual misconduct.^43^ Other entities such as Consumer Reports and the Informed Patient Institute have also pointed to the importance of clearly communicating this type of valuable information on board websites.^44^

SMBs can use social media to communicate with and educate the public about their function. Social media presence involving the routine posting of information about boards can further raise awareness and help boards remain on the radars of the public. Information shared with the public could include what to expect during a medical exam, what constitutes inappropriate behavior by a physician, and how to file a complaint with the board. If allowed by state law, disciplinary orders, criminal convictions, and other information should be made available. All information should be provided in a clear manner so that the public can understand and access it easily.

Beyond educating the public, SMBs can leverage relationships with professional organizations such as state medical societies, the American Board of Medical Specialties, and local branches of national associations like the American Cancer Society to communicate and disseminate information to hospital administrators and other leaders in healthcare. Frequent communication with and education of those who lead healthcare organizations can help these leaders better identify physicians who commit egregious wrongdoing and motivate appropriate and consistent reporting. The study of 280 cases of egregious wrongdoing found that at least 20% of cases involved an ignored report.^45^ Ignored reports create the opportunity for harmful physicians to continue practicing medicine. Developing a partnership between SMBs and healthcare leaders can help cultivate more transparent and routine information sharing with boards.

*Recommendation 3: Update SMB websites to include: 1) educational information for physicians including specific examples of disciplinary actions taken against physicians who violate the medical practice act, and 2) resources to support physician wellness and promote early intervention to mitigate and prevent behaviors that may increase the risk of egregious acts.*

All physicians should be familiar with their state’s medical practice act — the statute that defines the practice of medicine, licensing requirements, and oversight by state medical boards. Not all SMB websites have clear, easy-to-find links to their state medical practice acts. SMBs can use the board’s website, social media, and other online platforms to communicate with and educate physicians and other healthcare providers about the board’s function and medical practice acts. Beyond including this information on the website, boards can proactively reach out to their licensees with an introductory email that alerts licensees to the board website and provides licensees with links to the medical practice act and a place where licensees can sign up for board newsletters. Emails are efficient for communicating critical information and updates to licensees. Boards can also provide continuing education events at state medical and osteopathic conferences about board functions, standards for maintaining a license free of any disciplinary action, and the importance of and mechanisms for reporting peers who engage in wrongdoing. The role and function of SMBs could also be introduced in undergraduate and graduate medical education. These modes of communication can also be used to increase awareness about issues of importance for licensees, such as changes to state laws or board policies and standards.

SMBs can also provide physician education in the domains of professionalism and boundaries, using standardized, widely agreed-upon definitions rather than regional or state definitions whenever possible. Inter-generational differences among physicians suggest a need for clear and routine reminders about consistency in professionalism and the privilege of licensure. Norms and acceptable practices change. For example, at one time, gynecological exams were routinely performed on women under anesthesia without their consent.^46^ Though this practice continues today at some institutions, advocates are speaking out against nonconsensual exams.

SMBs are authorized by medical practice acts to establish professional standards. Because state statutes vary, SMBs should provide specific information about what constitutes unprofessional conduct under state law and typical examples of what disciplinary actions could be imposed for violations. Expectations for behavior and potential consequences for violations should be clear, consistent, and known to physicians, patients, board members, and other relevant decision-makers. Although most egregious wrongdoers are motivated by self-serving outcomes such as financial gain or sexual gratification,^47^ communicating with physicians about what will happen if they engage in misconduct could deter some potential wrongdoers from committing wrongdoing in the first place. As another means of deterring wrongdoing, each board website should make prior board actions publicly available if allowable by state law.

*Recommendation 4: Inform law enforcement that they can report accusations against a physician to the state medical board even if criminal charges are not filed.*

Providing information to and educating other stakeholders such as state and local law enforcement agencies about the board’s function are integral to identifying and stopping egregious wrongdoing by physicians. When boards have comprehensive information about criminal allegations, arrests, and charges, they can identify patterns of alleged behaviors by physicians that could signal a danger to the public. SMBs can educate law enforcement, including state and local law enforcement and the FBI, about the board’s function and the value of law enforcement and the information they can provide to board investigations. Boards can also coordinate with law enforcement to conduct investigations, even in the absence of a state statute mandating that law enforcement share information with the state medical board. While records can be subpoenaed, this may not always be helpful. Ideally, boards will want the cooperation of those who report to the police, which can further facilitate information sharing. Moreover, collaborating with law enforcement on investigations can help open lines of communication with complainants who may be willing to participate in the SMB disciplinary process.

Specifically, SMBs can request that police departments notify the board when there is an accusation of criminal behavior against a physician. SMBs may be able to assist law enforcement in determining whether charges should be filed. For example, police failed to charge Larry Nassar with sodomy after he was allowed to respond to questions about whether his activities were medically appropriate.^48^ Boards can confirm with law enforcement the accuracy of claims made by accused physicians, and this expertise can support the goals of law enforcement. Members of SMBs are more likely than law enforcement to have the expertise needed to accurately assess whether a physician’s conduct is medically appropriate or within the standard of care. In the Nassar case, the police took him at his word when he claimed to be using standard techniques, when in fact he was not.^49^ Notifying a SMB of accusations may also be appropriate even if the police investigation does not find enough evidence to arrest the physician or file criminal charges (e.g., probable cause). Unlike in criminal trials, many SMBs can determine that a violation occurred based on a preponderance of the evidence rather than proof beyond a reasonable doubt. SMBs can also determine that a violation of a medical practice act occurred even if civil or criminal charges are dropped.

Law enforcement can also educate boards about proper channels of communication based on the type of concern in question. For instance, if a board is investigating online threats and possible cyber security issues, it may be appropriate for boards to notify the FBI in addition to local law enforcement. Boards could create formal education programs for state and local police departments, and identify a point person or establish informal, or even formal, channels of communication regarding accusations against physicians. For coordination with law enforcement to be effective, boards should prioritize fostering good relationships with state and local law enforcement and strive for mutually beneficial partnerships. For example, relationship building could take the form of networking at professional functions (e.g., a Sheriff’s Association meeting).

## Cluster 3: Internal Board Operations and Investigations

*Recommendation 5: Implement a screening committee that triages incoming complaints.*

Up to 90% of complaints against physicians come from the public rather than from healthcare institutions.^50^ Due to the volume of complaints that SMBs receive, it is essential to have a system that allows for allegations of serious misconduct, such as accusations of sexual misconduct or other direct harms to patients, to be identified and investigated quickly to protect patients. A screening committee to triage incoming complaints can resolve minor cases (e.g., billing disputes that are not fraudulent) while quickly identifying those that pose an immediate threat to patient safety. As alluded to previously, it is important that the screening committee is comprised of diverse individuals and includes public members. No complaint regarding possible sexual misconduct should be dismissed without review by a screening committee trained in recognizing and mitigating the effects of implicit bias, that includes public members, and that is gender diverse.

Some screening committees are purely administrative in nature, which may be necessary given the number of reports made to boards in states with large patient populations. Boards with screening committees that have an administrative focus may want to shift toward more thorough and deliberative screening. Boards should also consider establishing and applying specific rules related to the escalation of complaints. For example, boards could establish a rule that requires certain reported actions, such as sexual misconduct, to always be escalated to a standing or ad hoc committee for urgent review so that these cases are not dismissed, deprioritized, or delayed. Establishing rules of this nature can be helpful to mitigate bias in deciding which complaints are escalated. These rules can also be especially helpful for small boards where establishing standing screening committees may not be feasible and where only one individual is responsible for screening reports.

*Recommendation 6: Investigate egregious wrongdoing that directly harms patients using specialized, diverse, and trauma-informed teams.*

The range of circumstances that benefit from trauma-informed teams provides a compelling reason for all board members to receive formal training in trauma-informed investigations and interactions.^51^ Trauma-informed care includes recognizing signs of trauma and responding to this trauma in a manner that does not re-traumatize; this includes adopting policies, procedures, and practices that incorporate trauma-specific knowledge.^52^ Educating board members about trauma can affect how investigations are handled and what decisions are made about opportunities for physician remediation and license reinstatement.^53^

Diverse and trauma-informed teams are especially important in investigations of sexual misconduct allegations. While sexual assault can happen to anyone, 91% of reported adult victims of rape and sexual assault are female.^54^ This figure may differ in cases involving children, with some sources citing nearly equal proportions of girls and boys.^55^ Because trauma can happen to anyone of any gender, diverse and trauma-informed teams should review cases involving sexual misconduct.^56^ Those who investigate these cases should be trained in the range of behaviors that constitute sexual abuse and violations, and on the effects of trauma. Trauma-informed teams can also be beneficial for investigating other cases of serious patient harm, such as physician negligence or unnecessary procedures that cause significant physical harm.

We recognize that not all boards have dedicated investigative teams. Some boards, especially boards with limited resources, have an individual who carries out investigations alone. We advocate for a team-based investigative approach but acknowledge that is not feasible for some boards. In cases where there is only one person investigating, these individuals should have others available as resources and know who to reach out to with questions or to confirm assumptions.

Regardless of the approach to investigations, all board members and staff, including board attorneys, should be educated on the effects of trauma. The work of investigators (e.g., building a case in the setting of trauma) may be compromised when board members adjudicate a case and do not understand trauma and the impact that trauma has on the complainant, including on the complainant’s memory and recall. Ensuring that everyone involved in investigative processes is trained to understand the effects of trauma was a major recommendation from the FSMB workgroup on physician sexual misconduct.^57^

*Recommendation 7: Provide examples of the types of alleged misconduct that prompt special actions to protect the public (e.g., consideration of emergency suspension of a license or expedited investigation).*

Typically, when a SMB determines the public’s health or safety is in imminent danger, it is authorized to take action by expediting an investigation, summarily suspending the physician’s medical license, or both.^58^ SMBs should clearly communicate the grounds for summary suspension under state law, including examples of specific behaviors that have satisfied this standard in the past. Providing specific, non-exclusive examples can make licensed physicians aware of, and appreciate the gravity of, the consequences of egregious wrongdoing.

Boards should be consistent in their standards for invoking an expedited investigation or emergency suspension. A consistent definition of what behaviors prompt special protections accomplishes several aims: 1) allows for greater and more comprehensive understanding by all stakeholders, including physicians under investigation, about when special procedures are warranted, 2) conveys a direct message to physicians about the consequences of egregious wrongdoing, and 3) fosters transparency about board standards and policies, which promote consistency in investigations and disciplinary actions.^59^ Boards can leverage consistent definitions and actions to track and evaluate trends, frequencies, and the impact of disciplinary actions over time.

As our team discussed Recommendation 7 and special actions that might take place, the notion of expedited remedial education emerged as an expansion of the initial recommendation. The opportunity for expedited remedial education interventions exists during an expedited investigation or even a summary suspension. If the licensee is under investigation, swift remediation prevents them from continuing their conduct of concern while still in practice but before any action is taken against their license. SMBs may wish to consider a category of practice and professional concern that does not rise to the level of imminent danger, but merits a “practice pause” until issues are addressed. If the licensee is under a summary suspension, they could benefit from remedial education, therapy, evaluation by a Physician Health Program (PHP), or other related activities even before a decision is made regarding their license.

## Cluster 4: Improve Coordination and Information Sharing Among Stakeholders

*Recommendation 8: Consistently provide complete disciplinary information to the FSMB Physician Data Center, which allows for disciplinary alerts to be sent to other jurisdictions in which the physician holds a license.*

The FSMB’s Physician Data Center (PDC) is a database of all United States SMBs’ disciplinary and licensure data.^60^ Most SMBs use the PDC but do so at varying frequencies. Although there are many reasons for inconsistent reporting,^61^ regular, consistent, and detailed reporting by SMBs should be part of the solution. SMBs should consistently update the PDC when they take disciplinary action against any physician. Ensuring that the PDC is comprehensive, up-to-date, and accurate provides healthcare entities with the ability to obtain information about a physician’s past behaviors and determine whether a physician poses a risk to patients. Tracking disciplinary and licensure data nationally also establishes patterns of behavior as physicians move to new medical institutions, practices, or across state lines.^62^

It should be noted that the PDC differs from, but is equally important compared to, the National Practitioner Data Bank (NPDB). The NPDB is a database of information on medical malpractice and adverse actions pertaining to healthcare providers and is another tool that SMBs should use to document and track physician behavior that may warrant disciplinary action.^63^ While the PDC and NPDB are not available to the public, hospitals and boards can access these databases. SMBs should also consistently report disciplinary actions to the NPDB in addition to the PDC, as the NPDB is the database that hospitals are required to check if they want antitrust immunity for privileges actions. Being consistent in reporting disciplinary actions to both the PDC and NPDB facilitates transparent information sharing with the various stakeholders who query these databases to make informed decisions about physician employment and licensure.

## Cluster 5: Licensing and Disciplinary Considerations

*Recommendation 9: Consider revocation when a physician repeatedly commits lesser acts of wrongdoing, especially following remedial efforts.*

The best predictor of future behavior is past behavior, unless there are appropriate and effective interventions. Repeat offenses indicate that there may be larger, more substantial issues with a physician’s conduct in the future.^64^ If permitted by state law, revoking a physician’s license after repeated or escalating acts of lesser wrongdoing that directly harm patients effectively signals intolerance of these behaviors. Furthermore, revocation can also prevent harm to patients. When physicians are found to have behaved egregiously, it is common to find that they have engaged in several other forms of wrongdoing.^65^ For example, sexual violations are nearly always preceded by boundary violations such as inappropriate comments or touching. While these behaviors may occur without later sexual violations, repeated instances demand close monitoring.

Escalation of sanctions is appropriate up to and including formal discipline and revocation, especially when bad behavior persists. This would require that SMBs keep well-organized and complete records of complaints, investigations, and “non-actions” so that they have the information needed to assess repeated minor violations. If boards establish the triage process recommended earlier, it should include collection, retention, and monitoring of complaint-related data. Emphasis should be on repeated offenses after training, warnings, or other prior remediation actions.

While boards should take “red flags” seriously, they should impose disciplinary measures in a manner consistent and commensurate with the severity and frequency of the offense in question. However, sanctions should not be trivial (e.g., a fine or warning letter with no recommended actions for cultivating behavior change), even for relatively minor offenses. Interventions such as remedial education, Physician Health Program involvement, practice monitoring, etc., should be imposed swiftly in the interest of public protection while the licensee is still in practice.

Rehabilitating and remediating licensee behavior must be done explicitly, with intention and consistency, and involve more than the mere passage of time. Boards should clearly operationalize what constitutes successful licensee rehabilitation and remediation and require the licensee to take full responsibility for all past actions. This might include: 1) explicitly acknowledging and documenting licensee wrongdoing to the extent possible even if the licensee was able to formally “plead down” a criminal charge or expunge their record, 2) requiring the licensee to work with professionals to identify and address the underlying root cause that led to the misconduct, and 3) mandating licensees to participate in ongoing “recovery” work with professional oversight.

*Recommendation 10: When physician behavior is found appropriate for discipline, bring the disciplinary recommendation to the full board for approval.*

Effective disciplinary action is commensurate with the type and magnitude of wrongdoing done by a physician, as well as other contextual factors. Lenient disciplinary action does not effectively deter wrongdoing. Bringing a disciplinary recommendation to the full board for approval ensures that an instance of wrongdoing is not being under-penalized (e.g., “a slap on the wrist”) and that standards are being consistently upheld. In many cases, physicians agree to a disciplinary recommendation without full board involvement, and this allows the action to be kept confidential; full board involvement where a board committee or panel takes an action ensures that the action becomes public. Full board approval of an order can also ensure that mitigating and aggravating factors are being weighed appropriately and that licensee-specific attributes, such as acceptance of responsibility and level of insight, are considered.

Disciplinary orders should include clear instructions about how to comply with stipulations as well as the necessary resources to fulfill them. To facilitate this process and ensure consistency, SMBs should compile a board-vetted list of pre-approved disciplinary actions, including remedial courses or course providers and assessment programs. In the interest of public safety, boards should consider shortening timeframes for completing remedial education from the typical 6-12 months to 60-90 days.

*Recommendation 11: Require all physicians to report any serious disciplinary action (e.g., suspension, probation, expulsion, being requested or allowed to resign in lieu of discipline) during medical school and residency training at the time of their application for licensure.*

Boards have myriad opportunities to adopt practices that can better protect the public from harmful physicians without the need for legislative or government action. These practices span several domains, including matters of board composition and training, board outreach and education, internal board operations, stakeholder engagement, and licensing and disciplinary considerations. We hope that the summary and synthesis of these select expert-informed recommendations can be a resource for boards as they seek to adopt new policies and practices that improve their efforts to better protect patients.

Behavior that results in disciplinary action during medical school is predictive of disciplinary action by SMBs later in a physician’s career.^66^ By requiring licensure applicants to report probationary and disciplinary action during medical school (including hospital actions and adverse employment actions from physician group practices) and sign a waiver that permits the board to verify the information reported, SMBs can identify physicians with a greater risk for future wrongdoing. The same could be done during residency. This is the practice followed by other professional licensure programs; for example, admission to the State Bar in every U.S. jurisdiction requires applications to meet character, fitness, and other qualifications in order to practice law.^67^ Collecting this information can help SMBs be more proactive in their approach to protecting patients.

When boards are made aware of disciplinary action during training, they can more proactively monitor physicians and take this information into account if later misconduct is reported and verified. Interstate medical licensure compact initiatives can further support proactive monitoring and information sharing about physician conduct among groups of SMBs.^68^ As mentioned in Recommendation 9, boards may consider license suspension or revocation when repeated instances of lesser acts of wrongdoing occur; however, such patterns may remain hidden when SMBs struggle to identify wrongdoing or complaints that occurred in other states.

Reporting unprofessional and problematic behavior in medical school and residency can be inconsistent. It is beyond the ability of boards to require medical schools to keep records and report to SMBs, but partnering with medical schools and encouraging them to report can help. Moreover, medical schools can support this partnership by communicating with medical trainees about the implications of errors, gaps, omissions, and dishonesty on a licensing application. In some states that currently require reporting of disciplinary action during medical school, an applicant’s failure to accurately and fully report this information can result in denial of licensure.^69^

## Conclusion

There is a growing awareness of the role SMBs have to play in protecting the public from egregious wrongdoing by physicians. Too many cases of patient abuse involve a large number of victims across a long period of time. SMBs are often in a position to change these circumstances when they establish and consistently utilize and enforce policies, procedures, and resources that are needed to impose severe disciplinary actions in a timely and fair manner. Many improvements in board processes require action by state legislatures, changes to state statutes, and increases to SMB budgets. While most of the actions we advocate in this paper would be facilitated and enhanced by existing or new statutes or regulations, and more frequently increased budgets, most of them can be at least partially implemented independently with modest budgetary impact in the short-term. The recommendations expanded upon in this paper are the result of input from individuals of various roles and expertise, including members of the FSMB, SMB members, health lawyers, patient advocates, and other healthcare leaders. Future efforts may wish to engage an even wider range of stakeholders on these topics, possibly with a greater emphasis on engaging patient and consumer advocates.

Boards have myriad opportunities to adopt practices that can better protect the public from harmful physicians without the need for legislative or government action. These practices span several domains, including matters of board composition and training, board outreach and education, internal board operations, stakeholder engagement, and licensing and disciplinary considerations. We hope that the summary and synthesis of these select expert-informed recommendations can be a resource for boards as they seek to adopt new policies and practices that improve their efforts to better protect patients.

## Note

This project was supported by funding from the Greenwall Foundation Making a Difference Program (PI: McIntosh). The authors thank all Delphi panelists for their participation in this project

## References

[1] Federation of State Medical Boards. U.S. Medical Regulatory Trends and Actions. Dallas, TX; May 2014; National Center for Health Statistics. Health, United States, 2016: With Chartb ook on Long-term Trends in Health, 2017; J.A. Harris and E. Byhoff , “Variations by State in Physician Disciplinary Actions by U.S. Medical Licensure Boards,” BMJ Quality & Safety 26, no. 3 (2017): 200–208; R.R. Peachman, “What You Don’t Know About Your Doctor Could Hurt You,” Consumer Reports, April 20, 2016.

[2] J.M. DuBois , E.E. Anderson , J.T. Chibnall , J. Mozersky , and H.A. Walsh , “Serious Ethical Violations in Medicine: A Statistical and Ethical Analysis of 280 Cases in the United States From 2008–2016,” The American Journal of Bioethics 19, no. 1 (2019): 16–34. 10.1080/15265161.2018.1544305 PMC6460481 30676904

[3] *Id.*; S.M. Wolfe , C. Williams , and A. Zaslow , Public Citizen’s Health Research Group Ranking of the Rate of State Medical Boards’ Serious Disciplinary Actions, 2009-2011 (Washington, DC: Public Citizen, 2012); S. Wolfe and R.E. Oshel, *Ranking of the Rate of State Medical Boards’ Serious Disciplinary Actions, 2017-2019* (Public Citizen’s Health Research Group, 2021).

[4] *Id.*

[5] *United States District Court Eastern District of Michigan Souther Division. United States of America v. Farid Fata*, 2017.

[6] L. Berman , “One Nurse’s Gutsy Effort to Protect Patients,” The Detroit News, Feb. 6, 2015, *available at* <https://www.detroitnews.com/story/opinion/columnists/laura-berman/2015/02/05/berman-gutsy-nurse-whistle-blower/22964301/> (last visited Dec. 21, 2023).

[7] A. Schecter , K. Nathanson , and J. Schuppe , “Farid Fata Gets 45 Years in Prison for Scamming Hundreds of Cancer Patients,” NBC News, July 10, 2015, *available at* <https://www.nbcnews.com/news/us-news/farid-fata-gets-45-years-prison-scamming-hundreds-cancer-patients-n389956> (last visited Dec. 21, 2023).

[8] D. Robbins, “Patients Report Abuse, But Doctor Still Practices,” *The Atlanta Journal-Constitution*, 2016, *available at* <https://doctors.ajc.com/texas_doctor_sex_abuse/?ecmp=doctorssexabuse_microsite_nav> (last visited Dec. 21, 2023).

[9] Texas State Board of Medical Examiners, In the Matter of The License of Philip J. Leonard, MD: Temporary Suspension Order2003 March 8.

[10] *Id.*

[11] *Id.*

[12] *Id.*

[13] *Id.*

[14] *Id.*

[15] Texas State Board of Medical Examiners, In the Matter of the Complaint Against Philip Joseph Leonard, MD: Agreed Order2004 December 10.13.

[16] *Id.*

[17] *Id.*

[18] D. Robbins , “Doctor Accused By 17 Females Loses License After Male Patient’s Accusation Of Sexual Impropriety,” Dayton Daily News, July 14, 2018.

[19] *Id.*

[20] *Id.*

[21] D. Grant and K.C. Alfred , “Sanctions and Recidivism: An Evaluation of Physician Discipline by State Medical Boards,” Journal of Health Politics, Policy and Law 32, no. 5 (2007): 867–885. 17855720 10.1215/03616878-2007-033

[22] *Id.*

[23] T. McIntosh , E. Pendo , H. Walsh , K. Baldwin , and J.M. DuBois , “Protecting Patients from Egregious Wrongdoing by Physicians: Consensus Recommendations from State Medical Board Members and Staff,” Journal of Medical Regulation 107, no. 3 (2021): 5–18.

[24] *Id.*

[25] T. McIntosh , E. Pendo , P.A. King , K.K. Dineen , J.D. Oliva , L.L. Broome , et al., “2021: Disciplining Physicians Who Inflict Harm: New Legal Resources for State Medical Board Members,” Journal of Health Law and Policy Symposia 1 (2021).

[26] Federation of State Medical Boards, *U.S. Medical Regulatory Trends and Actions* (Washington, DC; 2018); Federation of State Medical Boards of the United States, U.S. Medical Regulatory Trends and Actions 2018, Federation of State Medical Boards of the United States; 2018.

[27] FSMB Workgroup on Physician Sexual Misconduct, “Report and Recommendations of the FSMB Workgroup on Physician Sexual Misconduct,” *Journal of Medical Regulation* 106, no. 2 (2020): 17-36.

[28] FSMB Statement on Diversity, Equity, and Inclusion in Medical Regulation and Health Care, Press Release, Apr 15, 2021; L.L. Broome and J.M. Conley , “Diversity from the Perspective of Corporate Boards and Lawyer Disciplinary Boards,” Saint Louis University Journal of Health Law and Policy 15, no. 1 (2021): 121–150.

[29] S. Sommers , “On Racial Diversity and Group Decision Making: Identifying Multiple Effects of Racial Composition on Jury Deliberations,” Journal of Personality and Social Psychology 90 (2006): 597–612; V. Hunt, D. Layton, and S. Prince, “Diversity Matters,” *McKinsey & Company* 1, no. 1 (2015): 15-29; K.W. Phillips, “How Diversity Makes Us Smarter,” *Scientific American* 311, no. 4 (2014): 43-47. 16649857 10.1037/0022-3514.90.4.597

[30] D. Rock and H. Grant, “Why Diverse Teams are Smarter?” *Harvard Business Review*, November 4, 2016, *available at* <https://hbr.org/2016/11/why-diverse-teams-are-smarter> (last visited Dec. 21, 2023); Y. Nili, “Beyond the Numbers: Substantive Gender Diversity in Boardrooms,” *Indiana Law Journal* 94 (2019): 145.

[31] *Id.*; L. Hong and S.E. Page , “Groups of Diverse Problem Solvers Can Outperform Groups of High-Ability Problem Solvers,” Proceedings of the National Academy of Sciences of the United States of America 101, no. 46 (2004): 16385–16389. 15534225 10.1073/pnas.0403723101 PMC528939

[32] *Id.*

[33] *Id*.

[34] *Id.*

[35] Federation of State Medical Boards, *Guidelines for the Structure and Function of a State Medical and Osteopathic Board: FSMB*, *available at* <https://www.fsmb.org/siteassets/advocacy/policies/guidelines-for-the-structure-and-function-of-a-state-medical-and-osteopathic-board.pdf> (last visited Dec. 21, 2023).

[36] *Id*.

[37] A. AbuDagga , S.M. Wolfe , M. Carome , and R.E. Oshel , “Cross-Sectional Analysis of the 1039 U.S. Physicians Reported to the National Practitioner Data Bank for Sexual Misconduct, 2003-2013,” PLoS One 11, no. 2 (2016): 1–13; K.S. Arora, S. Douglas, and S.D. Goold, “What Brings Physicians to Disciplinary Review? A Further Subcategorization,” *AJOB Empirical Bioethics* 5, no. 4 (2014): 53-60; Federation of State Medical Boards, “Report and Recommendations of the FSMB Workgroup on Physician Sexual Misconduct,” *Journal of Medical Regulation* 106, no. 2 (2020): 17-36. 10.1371/journal.pone.0147800 PMC4739584 26840639

[38] I.N. Onyeador , S-kT. Hudson , and N.A. Lewis , Jr., “Moving Beyond Implicit Bias Training: Policy Insights for Increasing Organizational Diversity,” Policy Insights from the Behavioral and Brain Sciences 8, no. 1 (2021): 19–26; C. FitzGerald, A. Martin, D. Berner, and S. Hurst, “Interventions Designed to Reduce Implicit Prejudices and Implicit Stereotypes in Real World Contexts: A Systematic Review,” *BMC Psychology* 7, no. 1 (2019): 1-12. 10.1186/s40359-019-0299-7 PMC6524213 31097028

[39] “FSMB Launches Task Force on Health Equity and Medical Regulation,” press release, March 31, 2021.

[40] Harris Insights & Analytics, *Harris on Demand, The Harris Poll: State Medical Boards Awareness Study*, *available at* <https://www.fsmb.org/siteassets/advocacy/news-releases/2018/harris-poll-executive-summary.pdf> (last visited Dec. 21, 2023).

[41] E.D. Solomon , H. Walsh , M. Parsons , T. McIntosh , J. Mozersky , and J.M. DuBois , “The Development of a Resource to Help Patients Receive Appropriate Care,” Journal of Health Care for the Poor and Underserved 32, no. 4 (2021); T. McIntosh, H. Walsh, M. Parsons, E.S. Solomon, J. Mozerskly, and J.M. DuBois, “Responding to Sexual Abuse in Healthcare: The Development of a Guide For Patients,” *Journal of Patient-Centered Research and Reviews* 9, no. 2 (2022).

[42] Federation of State Medical Boards. Social Media and Electronic Communications, Report and Recommendations of the FSMB Ethics and Professionalism Committee, 2019.

[43] N.K. Gartrell , N. Milliken , W.H. Goodson III , S. Thiemann , and B. Lo , “Physician-Patient Sexual Contact. Prevalence and Problems,” The Western Journal of Medicine 157, no. 2 (1992): 139–143; A. Judd, “Prestige Protects Even the Worst Abuser*,” Atlanta Journal-Constitution*, December 14, 2018; D. Robbins, “Repeat Offender Still Licensed to Treat Georgia Patients,” *The Atlanta Journal-Constitution*, 2016, *available at* <https://doctors.ajc.com/georgia_doctor_sex_abuse/?ecmp=doctorssexabuse_microsite_stories> (last visited Jan. 12, 2024); L. Norder, J. Ernsthausen, and D. Robbins, “Why Sexual Misconduct is Difficult to Uncover,” *Atlanta Journal-Constitution*, December 18, 2018, *available at* <http://doctors.ajc.com/sex_abuse_numbers/> (last visited Dec. 22, 2023); A. Hart, “Which Doctors Are Sexually Abusive?” *The Atlanta Journal-Constitution*, 2016, *available at* <https://doctors.ajc.com/doctors_who_sexually_abuse/> (last visited Jan. 12, 2024). 1441462 PMC1011231

[44] Consumers Union, The Informed Patient Institute. Medical Board Website Ratings 2016, *available at* <https://advocacy.consumerreports.org/wp-content/uploads/2016/03/Chart-website-review-CR-blobs-all-states-FINAL-4.pdf> (last visited Jan. 12, 2024); Consumer Reports, “Hawaii Rated Among Worst In Nation For Failing to Keep Patients Informed About Bad Doctors,” March 29, 2016, *available at* <https://advocacy.consumerreports.org/press_release/hawaii-rated-among-worst-in-nation-for-failing-to-keep-patients-informed-about-bad-doctors/> (last visited Dec. 22, 2023); Consumer Reports, “New Mexico Rated Among Worst In Nation For Failing to Keep Patients Informed About Bad Doctors,” March 29, 2016, *available at* <https://advocacy.consumerreports.org/press_release/new-mexico-rated-among-worst-in-nation-for-failing-to-keep-patients-informed-about-bad-doctors/> (last visited Dec. 22, 2023); Consumer Reports, “Utah Rated Among Worst In Nation For Failing to Keep Patients Informed About Bad Doctors,” March 29, 2016, *available at* <https://advocacy.consumerreports.org/press_release/utah-rated-among-worst-in-nation-for-failing-to-keep-patients-informed-about-bad-doctors/> (last visited Dec. 22, 2023); Consumer Reports, “Alaska Rated Among Worst In Nation For Failing to Keep Patients Informed About Bad Doctors,” March 29, 2016, *available at* <https://advocacy.consumerreports.org/press_release/alaska-rated-among-worst-in-nation-for-failing-to-keep-patients-informed-about-bad-doctors/> (last visited Dec. 22, 2023); Consumer Reports, “Wyoming Rated Among Worst In Nation For Failing to Keep Patients Informed About Bad Doctors,” March 29, 2016, *available at <* https://advocacy.consumerreports.org/press_release/wyoming-rated-among-worst-in-nation-for-failing-to-keep-patients-informed-about-bad-doctors/> (last visited Dec. 22, 2023); Consumer Reports, “Montana Rated Among Worst In Nation For Failing to Keep Patients Informed About Bad Doctors,” March 29, 2016, *available at* <https://advocacy.consumerreports.org/press_release/montana-rated-among-worst-in-nation-for-failing-to-keep-patients-informed-about-bad-doctors/> (last visited Dec. 22, 2023); Consumer Reports, “Indiana Rated Among Worst In Nation For Failing to Keep Patients Informed About Bad Doctors,” March 29, 2016, *available at* <https://advocacy.consumerreports.org/press_release/indiana-rated-among-worst-in-nation-for-failing-to-keep-patients-informed-about-bad-doctors/> (last visited Dec. 22, 2023); Consumer Reports, “Mississippi Rated Worst In Nation For Failing to Keep Patients Informed About Bad Doctors,” March 29, 2016, *available at* <https://advocacy.consumerreports.org/press_release/mississippi-rated-worst-in-nation-for-failing-to-keep-patients-informed-about-bad-doctors/> (last visited Dec. 22, 2023); Consumer Reports, “Consumers Union Urges Lawmakers to Support Bill Requiring Doctors on Probation for Serious Offenses to Notify Patients,” April 11, 2016, *available at* <https://advocacy.consumerreports.org/press_release/consumers-union-urges-lawmakers-to-support-bill-requiring-doctors-on-probation-for-serious-offenses-to-notify-patients/> (last visited Dec. 22, 2023).

[45] *Id.*

[46] E. Ching , P. Yao , Y. Jaffe , and A. Chan , “Consent for the Pelvic Examination Under Anesthesia by Medical Students: Recommendations by the Association of Professors of Gynecology and Obstetric,” Obstetrics & Gynecology 135, no. 4 (2020): 973. 10.1097/AOG.0000000000003796 32217957

[47] *Id.*; J.M. DuBois , J.T. Chibnall , E.E. Anderson , H.A. Walsh , M. Eggers , K.A. Baldwin , et al., “Exploring Unnecessary Invasive Procedures in the United States: A Retrospective Mixed-methods Analysis of Cases from 2008-2016,” Patient Safety in Surgery 11, no. 1 (2017): 30; J.M. DuBois, H.A. Walsh, J.T. Chibnall, E.E. Anderson, M.R. Eggers, M. Fowose, et al., “Sexual Violation of Patients by Physicians: A Mixed-Methods, Exploratory Analysis of 101 Cases. Sex Abuse,” 31, no. 5 (2019): 503-523; J.M. DuBois, J.T. Chibnall, E.E. Anderson, M. Eggers, K.A. Baldwin, M. Vasher, “A Mixed-Method Analysis of Reports on 100 Cases of Improper Prescribing of Controlled Substances,” *Journal of Drug Issues* 46, no. 4 (2016): 457-472.29270224

[48] D. Eggert, “Judge Says 265 People Have Come Forward to Say They Were Victims of Disgraced Former Sports Doctor Larry Nassar,” *Chicago Tribune*, January 31, 2018, *available at* <http://www.chicagotribune.com/sports/international/ct-larry-nassar-more-sentencing-20180130-story.html> (last visited Dec. 22, 2023); K. Howley, “Everyone Believed Larry Nassar The Predatory Trainer May Have Just Taken Down USA Gymnastics. How Did He Deceive So Many For So Long?” The Cut, November 19, 2018, *available at* <https://www.thecut.com/2018/11/how-did-larry-nassar-deceive-so-many-for-so-long.html> (last visited Dec. 22, 2023).

[49] A. Pesta , The Girls: An All-American Town, a Predatory Doctor, and the Untold Story of the Gymnasts who Brought Him Down (Seal Press, 2019).

[50] *Id.*

[51] P.A. King , E. Gerard , M. Staz , and E.M. Fish , “Contextualizing and Strengthening State Medical Board Responses to Physician Sexual Misconduct,” Saint Louis University Journal of Health Law and Policy 15, no. 1 (2021): 151–182.

[52] U.S. Department of Health and Human Services. SAMHSA’s concept of trauma and guidance for a trauma-informed approach. HHS Publication No. (SMA) 14-4884; 2014.

[53] *Id*.

[54] C. M. Rennison , Rape and Sexual Assault: Reporting to Police and Medical Attention, 1992-2000 (Washington, DC: U.S. Department of Justice, Office of Justice Programs, 2002).

[55] Statista Research Department, “Number of Child Abuse Cases in the United States in 2019, by Gender of the Victim,” 2021.

[56] Federation of State Medical Boards, “Physician Sexual Misconduct,” Washington, DC, 2020.

[57] *Id*.

[58] *Id*.

[59] *Id*.

[60] Federation of State Medical Boards, “Physician Data Center,” Washington, DC, 2021, *available at* <https://www.fsmb.org/PDC/> (last visited Dec. 22, 2023).

[61] *Id*.

[62] Federation of State Medical Boards, FSMB Certifications, Washington, DC, 2021, *available at* <https://www.fsmb.org/about-fsmb/fsmb-certifications/> (last visited Dec. 22, 2023).

[63] U.S. Department of Health & Human Services, NPDB National Practitioner Data Bank Chantilly, VA, 2021, *available at* <https://www.npdb.hrsa.gov/topNavigation/aboutUs.jsp> (last visited Dec. 22, 2023).

[64] *Id*.

[65] *Id*.

[66] M.A. Papadakis , C.S. Hodgson , A. Teherani , N.D. Kohatsu , “Unprofessional Behavior in Medical School is Associated with Subsequent Disciplinary Action by a State Medical Board,” Academic Medicine: Journal of the Association of American Medical Colleges 79, no. A3 (2004): 244–249. 14985199 10.1097/00001888-200403000-00011

[67] University of Houston Law Center, “Character and Fitness,” Houston, TX, 2020, *available at* <https://www.law.uh.edu/admissions/apply-now-character-and-fitness.asp> (last visited Dec. 22, 2023); American Bar Association, “2020-2021 Standards and Rules of Procedure for Approval of Law Schools,” Chicago, IL, 2020, *available at* <https://www.americanbar.org/groups/legal_education/resources/standards/> (last visited Dec. 22, 2023).

[68] B.T. Maresh , “The Interstate Medical Licensure Compact: Making the Business Case,” Journal of Medical Regulation 100, no. 2 (2014): 8–27

[69] Federation of State Medical Boards*, The Medical Licensing Process: A Module for Medical Students and Residents*, 2021, *available at* <https://www.fsmb.org/education/the-medical-licensing-process/> (last visited Dec. 22, 2023).

